# Improving Reliability and Explainability of Medical Question Answering Through Atomic Fact-Checking in Retrieval-Augmented Large Language Models: Creation and Validation Study

**DOI:** 10.2196/92090

**Published:** 2026-09-21

**Authors:** Juraj Vladika, Annika Domres, Mai Nguyen, Rebecca Moser, Jana Nano, Felix Busch, Lisa Adams, Keno K Bressem, Denise Bernhardt, Stephanie E Combs, Kai Borm, Florian Matthes, Jan C Peeken

**Affiliations:** 1 Department of Computer Science, TUM School of Computation Information and Technology, Technical University of Munich, Garching, Bavaria, Germany; 2 Department of Radiation Oncology, TUM University Hospital Rechts der Isar, TUM School of Medicine and Health, Technical University of Munich, Ismaninger Str. 22, Munich, Bavaria, Germany, 49 089 4140-4501; 3 Department of Diagnostic and Interventional Radiology, TUM University Hospital Rechts der Isar, TUM School of Medicine and Health, Technical University of Munich, Munich, Germany; 4 National Center for Tumor Diseases West, Essen, Germany; 5 Institute of Artificial Intelligence in Medicine, University Hospital Essen, Essen, Germany; 6 Department of Diagnostic and Interventional Radiology and Neuroradiology, University Hospital Essen, Essen, Germany; 7 Institute of Radiation Medicine (IRM), Helmholtz Zentrum München (HMGU), Munich, Bavaria, Germany; 8 German Consortium for Translational Cancer Research (DKTK), Partner Site Munich, Munich, Bavaria, Germany

**Keywords:** large language model, LLM, retrieval-augmented generation, RAG, fact-checking, atomic fact-checking, atomic fact, open-source, autoevaluation, hallucination, backtracing, prompt engineering, radiation oncology, medical fine-tuned, rubrics, question and answer, medical Q&A

## Abstract

**Background:**

Large language models (LLMs) exhibit extensive medical knowledge but are prone to hallucinations and show low fact-level explainability, limiting clinical adoption and regulatory compliance. Existing approaches, such as retrieval-augmented generation, partially address these issues by grounding answers in source documents; however, the aforementioned problems persist.

**Objective:**

We propose the application of an atomic fact-checking framework designed to enhance the reliability and explainability of LLMs in medical long-form question answering. By decomposing generated answers into discrete atomic facts and verifying each against an authoritative knowledge base of medical guidelines, this approach enables precise identification and correction of incorrect statements, alongside explicit linkage to supporting literature.

**Methods:**

The fact-checking algorithm operates within a retrieval-augmented generation framework: LLM-generated answers are decomposed into atomic facts (smallest and self-contained information units), each of which is assessed and corrected if FALSE. To determine an optimal strategy, the validation–question and answer (Q&A) set on prostate cancer treatment was tested under varying instructions. An extensive evaluation, including multireader assessments by human medical experts and the automated open Q&A benchmark AMEGA (Autonomous Medical Evaluation for Guideline Adherence), was conducted for the final pipeline. In addition to another radiooncologic test–Q&A set, anonymized real-world tumor board cases and an independent, established neurology-Q&A set were used. Given their transparency and accessibility advantages, we compared various open-source models in pairs of generalist models and their medical fine-tuned counterparts, with regard to performance and improvements by fact-checking.

**Results:**

The framework significantly reduced hallucinations and inaccuracies. Medical expert assessment and automated benchmarks demonstrated significant improvements in factual accuracy, achieving up to a 50% overall answer improvement and an 80% hallucination detection rate. Notably, the observed gain was strongest in real tumor-board questions—the most challenging dataset. Additionally, the framework achieved high explainability by tracing each atomic fact back to the most relevant chunks from the database, providing a granular, transparent explanation of the generated responses.

**Conclusions:**

To conclude, we present the application of an atomic fact-checking algorithm to medical Q&A. It identifies factual inaccuracies and hallucinations in LLM-generated answers, achieving the greatest gains on clinically realistic, complex questions. Correction via fact-checking improves the overall answer quality while achieving fact-wise explainability, paving the way for more credible clinical use of LLMs.

## Introduction

Large language models (LLMs) exhibit extensive medical knowledge [1]. However, LLMs are prone to hallucinations that may lead to harmful medical advice, and their often inaccurate citations reduce overall explainability [2]. This limits clinical use and complicates medical product certifications [3]. Current methods, such as retrieval-augmented generation (RAG), partially address these issues by grounding answers in source documents [4].

In RAG, the most common setup is to divide source texts into discrete chunks, embed them into a vector space, and retrieve as needed to ground LLM responses in updatable, authoritative information. Prior research shows that RAG can improve answer quality in medical question and answer (Q&A) [5]. Nevertheless, answers can still contain hallucinations, that is statements that are factually incorrect and contradict established knowledge. Additionally, fact-by-fact explainability of answers remains low, especially for complex medical queries. Existing approaches do not address the need for validating, backtracing, and correcting each individual claim within a long-form response [6,7].

Methods to increase the factuality of LLM outputs can involve additional pretraining or fine-tuning of models, which are computationally expensive and impossible for closed-source models. Hence, post hoc methods are emerging, where LLMs self-correct their responses only after they are generated [8]. A promising method is automated fact-checking, which detects individual facts from the generated response that contain information contradicting the authoritative knowledge, then rewrites these facts and the final response using correct information. Originating from journalism, where it is performed manually, fact-checking is increasingly used for hallucination correction [9,10]. However, existing approaches mostly focus on the encyclopedic and news domains and are underexplored for medical applications [11].

We define an atomic fact as the smallest self-contained and verifiable unit of information in a response generated by an LLM [10] (eg, “Trastuzumab is indicated for HER2-positive breast cancer”). We developed a framework that decomposes responses into atomic facts, each of which is independently verified against an authoritative vector database. This approach enables targeted correction of errors and direct tracing to the source literature, thereby improving the factual accuracy and explainability of medical Q&A. To the best of our knowledge, this is the first application of such an “atomic fact-checking” approach to the medical domain.

Beyond the provision of correct, up-to-date information, which represents a fundamental prerequisite for clinical adaptation, successful adoption of LLMs also depends on the users’ trust in both the generated answer and the underlying technical infrastructure.

Increasing scientific attention is being directed toward open-source models, resulting in capability improvements approaching those of proprietary models. This trend is driven by several advantages of open-source approaches, including greater transparency and enhanced data privacy, which favor their use [12]. Accordingly, we evaluated a variety of open-source models with respect to their performance and the improvements through fact-checking.

We hypothesize that (1) integrating an atomic fact-checking framework into an RAG pipeline improves the factual quality of medical Q&A responses while enabling fact-level traceability; (2) the magnitude of improvement achieved via fact-checking depends on the underlying model; and (3) atomic fact-checking yields performance improvements across different medical use cases, including real-world tumor board cases.

## Methods

### Atomic Fact-Checking Framework

**Figure 1.**
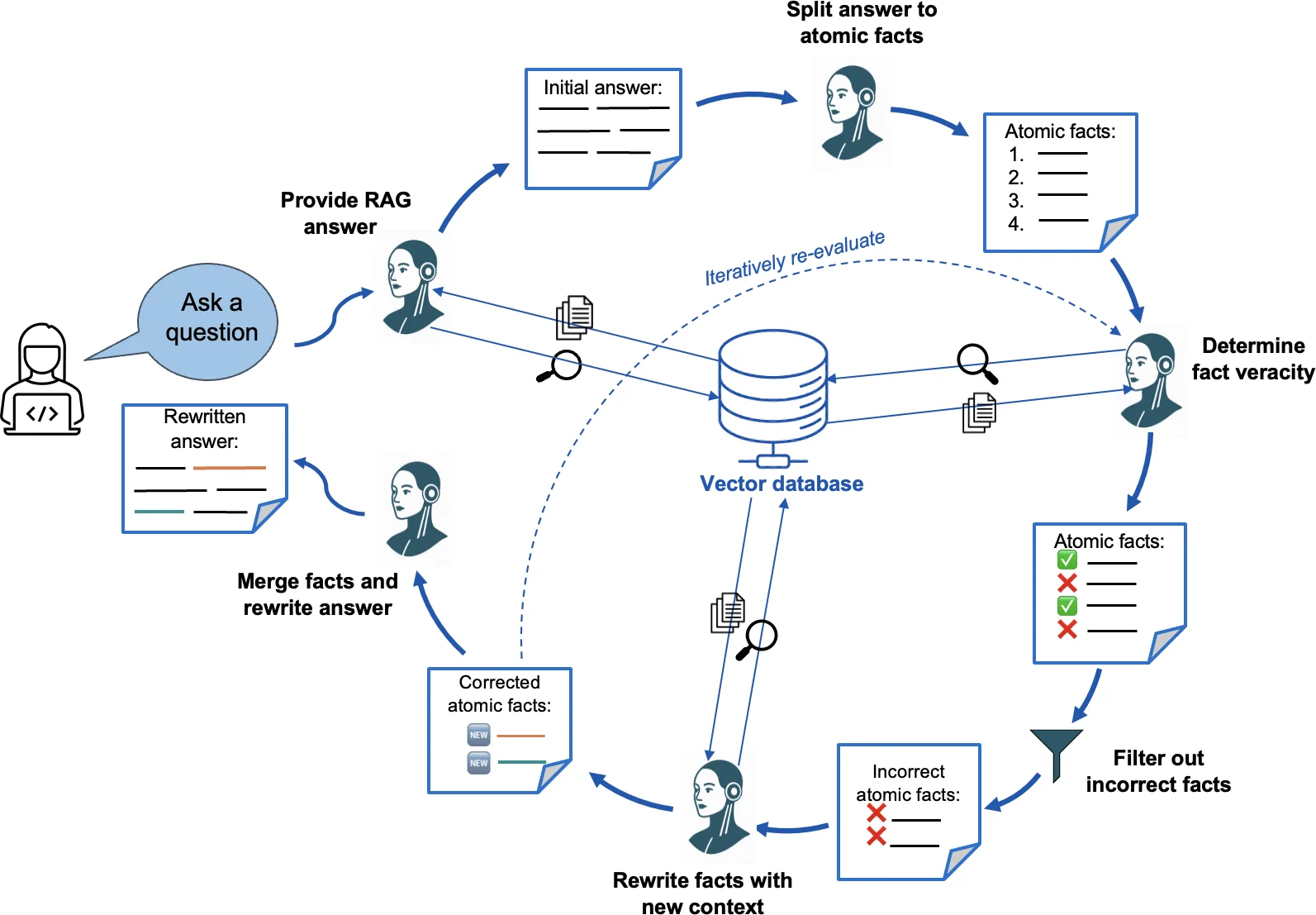
Architecture diagram illustrating the atomic fact-checking process. RAG: retrieval-augmented generation.

The LLM-TRIPOD (Transparent Reporting of a Multivariable Prediction Model for Individual Prognosis or Diagnosis) checklist is provided as Checklist 1. Our fact-checking framework consists of five steps, as shown in Figure 1: (1) generate an initial RAG-based response to the question; (2) split the response into atomic facts; (3) determine the veracity of each fact (categories: “TRUE” and “FALSE”) based on newly retrieved chunks; (4) rewrite the facts detected as incorrect and loop through steps 3 and 4 until all facts are correct, or for a maximum of 3 iterations; (5) rewrite the full response by incorporating the rewritten facts.

All 5 steps are implemented using dedicated LLM prompts, provided in Table S1 of Multimedia Appendix 1. For steps 1 to 4, in-context learning with 4 expert-annotated examples per prompt is used to enforce consistent atomic fact extraction and verdicting. All prompts and examples were created by a medical expert (JCP, 9 y of experience in radiation oncology). Prompts were refined until the performance was deemed satisfactory on a validation set; this included increasing the number of few-shot examples from 0 to 1 and finally to 4. The number of retrieved chunks was set to 7 because in the initial testing it provided the best balance between sufficient useful information and minimizing the noise from irrelevant chunks. An analysis using different numbers of top *k* chunks (3, 5, 7, and 10) is shown in Table S12 of Multimedia Appendix 1.

The knowledge base consisted of curated oncological guideline documents for prostate and breast cancer (see Table S5 in Multimedia Appendix 1). All PDF documents were converted into plain text using the open-source library PaperMage, which works well with visually rich scientific documents. The text was chunked into overlapping segments of 512 tokens (100-token overlap) and embedded using S-PubMedBERT [13], a transformer model pretrained on PubMed abstracts, giving it an increased semantic understanding of medical concepts, making it highly suitable for our medical use case. Chunks were stored in a ChromaDB vector database. For evidence retrieval, cosine similarity was used to select the 7 most relevant chunks for the question (in step 1) and then the 7 most similar ones for each atomic fact (step 3). All LLM generations were performed using GPT-4o (gpt-4o-2024-11-20; OpenAI) via the OpenAI API with a temperature set to 0 to ensure more deterministic outputs and reproducibility.

While the knowledge base serves as the primary source for both answer creation and verification, the input question itself is also incorporated as a reference source during the fact-checking process. This allows for the verification of patient-specific information included in the query that is not represented in guideline documents. Consequently, patient-specific facts are not incorrectly classified as FALSE only because they cannot be validated against the external knowledge base.

Two additional components of the pipeline were tested. Looping, where the results of one full fact-checking run (with atomic fact corrections) were used as the input to another full run, was tested to determine if it helps correct the facts that were still incorrect. Ensembling, where the fact-veracity prediction (“TRUE” or “FALSE”) was based not on just one prediction output but on multiple predictions using the same LLM and prompt, was used to determine whether fact-veracity prediction could be improved.

### Q&A Benchmark

The main task of this study was long-form medical question-answering. Two Q&A datasets were constructed by a medical expert (JCP), with each dataset comprising distinct questions about the diagnosis and treatment of prostate or breast cancer. Questions were based on the guideline content provided by the RAG system. They were categorized as fact-based (direct guideline knowledge) or patient-based (clinical vignettes), with varying complexity. To select the optimal prompting strategy, the first set, a validation set of 50 Q&A pairs on prostate cancer, was used. The final configuration was tested on the second set, a separate test set of 60 Q&A pairs (30 prostate and 30 breast cancer).

Asking questions on specialties other than prostate cancer, as in the validation set (implemented breast cancer Q&A in the test set and an independent, established neurology-Q&A set [2] of 65 Q&A items), ensured that no overfitting occurred.

To assess the framework in a realistic clinical setting, 40 anonymized real patient cases from a multidisciplinary tumor board were included. A total of 215 Q&As were used, all of which were checked by human expert evaluators.

All Q&A sets are available in the GitHub (Microsoft) repository [14]. An overview of all Q&As used and their respective questions is available in Table S10 of the Multimedia Appendix 1.

### Human Evaluation

Fact-checking performance was evaluated by comparing assigned verdicts (“TRUE” or “FALSE”) against human assessments. Human evaluation involved verifying the correctness of each atomic verdict and categorizing errors as hallucinations, incorrect information, missing information, or lack of context. Each atomic fact correction itself was analyzed for correctness, while undetected incorrect facts were considered false negatives. Final overall answers were compared to initial responses to determine whether fact-checking led to improvement, deterioration, or no change.

Validation and tumor board analyses were conducted by one medically trained scientist (AD), supervised by JCP. Test-set evaluation used four independent blinded physician raters (AD, FB, JN, and MN), with majority voting; disagreements (2 vs 2) were resolved by a blinded fifth rater (JCP). All four raters agreed in 85% of cases, with ties in 4% of cases. The Fleiss κ and Krippendorff α were both 0.16: this score is an artifact of unbalanced labels—since the LLM classifier was highly accurate, the “1” (correct) label was highly prevalent, making the “agreement expected by chance” very high.

The following metrics were computed: true positive (verdict “FALSE” confirmed by a human), true negative (verdict “TRUE” confirmed by a human), false positive (verdict “FALSE” not confirmed by a human), and false negative (verdict “TRUE” not confirmed by a human). Standard confusion matrix metrics for classification tasks (sensitivity, specificity, precision, *F*_1_, and accuracy) were calculated. Annotator guidelines are available in Multimedia Appendix 1.

### Auto Evaluation

The framework was further evaluated on the AMEGA (Autonomous Medical Evaluation for Guideline Adherence) benchmark [15], which tests adherence of LLMs to medical guidelines across 20 clinical domains. This benchmark comprises 20 patient cases (6‐8 questions each; 135 in total), with 1337 predefined evaluation criteria that can be used for automatic evaluation of generated answers. In the original study, responses were improved by iterative “question reasking”. Here, we applied our atomic fact-checking pipeline and compared the initial RAG answers with the corrected answers using the same criteria. The auto-evaluation was done using GPT-4o (gpt-4o-2024-11-20), and for each case, the relevant medical guidelines served as the retrieval database.

### Comparison of Open-Source Generalist vs Medical Models

Our experiments focused on differences in the fact-checking performance between medical fine-tuned, open-source LLMs and their counterpart general-purpose (ie, nonmedical) models. The compared models include Gemma 3 27B (Google Deepmind) vs MedGemma 27B (Google), Llama 3 70B (Meta AI) vs OpenBioLLM 70B (Saama AI Labs), and Qwen 3 32B (Alibaba Cloud) vs Qwen 3 Medical 32B (Alibaba Cloud). The validation set (50 prostate Q&A pairs) was used, and a human evaluation was conducted as in previous experiments.

In addition to the quantitative fact-checking evaluation, we performed a qualitative automated evaluation. Using the LLM-as-a-judge technique, we defined 7 rubrics that evaluated different aspects of the generated answers’ quality. We used GPT-4.1 as an independent judge model, which produced a numeric score (range 0‐1) for each answer. Each rubric used finely crafted evaluation criteria and steps, which were based on common metrics from a related LLM-as-a-judge framework, G-Eval [16] and adjusted by a medical expert (JCP) where needed. The rubrics used were correctness, completeness, clarity, context faithfulness, coherence, medical harmfulness, and calibration. Evaluation steps are listed in Table S8 of Multimedia Appendix 1.

### Baseline Approach

In order to evaluate how well our atomic fact-checking framework performs compared to other methods, we compared it with the popular framework self-refine [17]. In this framework, the main idea is to first generate an initial response using an LLM, then use the same LLM to provide feedback on how to improve the response, and finally use the same LLM to refine the response based on that feedback. We used the prompts from the original paper, slightly adapted for the Q&A use case (prompts can be found in Table S4 of Multimedia Appendix 1). We evaluated the approach on the AMEGA benchmark using GPT-4o, GPT-4o-mini, Gemma 3 27B, MedGemma 27B, and Llama 3.2 3B.

### Ethical Considerations

Institutional review board approval was obtained from the Institutional Review Board of the University Hospital of the Technical University of Munich (ethics approval number 2023‐626_1 S-NP). All patients were treated after obtaining informed consent. Additional informed consent for the scientific study was not necessary due to local legislation (Bayerisches Krankenhausgesetz [18,19]). All patient data were fully anonymized. There was no participant compensation.

## Results

### Architectural Structure

Across all evaluation sets, answers were split into a median of 7 (IQR 5‐8.75) atomic facts, yielding a total number of 428, 404, 519, and 474 facts for the validation (50 Q&A), testing (60 Q&A), tumor board (40 Q&A), and neurology (65 Q&A) sets, respectively. Evaluation on the validation set revealed the best architectural strategy as 4-shot prompting for answer generation, verdicting, and fact rewriting (ablation study: Table S7 and Figure S1 in Multimedia Appendix 1). In the atomic fact correction step, retrieving new chunks for each fact, instead of using the initial chunks (used for Q&A) for fact-checking and rewriting, increased the overall performance (both precision and sensitivity).

Looping through all facts labeled as “FALSE” further increased the true positive rate and positive predictive value while reducing falsifications in rewritten facts. The average false positive rate over 3 evaluation sets decreased from 2% to 1% to 0% throughout 3 iterations of the correction loop. Therefore, 3 iterations were chosen as the maximum, since this was enough in experiments to correct all unsupported facts. Ensembling for atomic veracity prediction and changes in temperature during ensembling did not yield a significant gain in performance (Figures S2 and S3 in Multimedia Appendix 1). While it slightly increased the overall *F*_1_-score, it also reduced sensitivity, the most important metric in our system. Therefore, our final pipeline uses 4-shot examples and 3-step looping but no veracity prediction ensembling.

Adding few-shot examples to LLM prompts decreased the precision scores (95, 71, and 61 for 0, 1, and 4-shot settings) but increased the sensitivity (recall) scores (44, 62, and 78). Sensitivity was preferred because it is more important in our system to detect any hallucinations, while any false positives could be dismissed during the rewriting phase.

### Fact-Checking Impact in Numbers and Sets

In the validation-Q&A and test-Q&A sets, this final framework achieved balanced accuracy scores of 87% and 74%, with hallucination detection of 50% and 38% and inaccuracy detection of 58% and 50%, respectively (Table 1). In the more complex tumor board test set, 25% of hallucinations and 24% of inaccuracies were found, with a balanced accuracy of 72%. The positive predictive value for false fact identification was 66%, 100%, and 85% for the validation, test, and tumor board sets, respectively.

**Table 1.** Human evaluation on 4 benchmark datasets. We present results for different metrics, as rated by medical expert annotators, across our 3 constructed and 1 external question and answer (Q&A) datasets.

Evaluation metric	Validation set^a^	Test set^a^	Tumor board^a^	Neurology Q&A^a^ [2]
Sensitivity (recall)	78	47	46	52
Specificity	96	100	99	100
Precision (PPV^b^)	66	100	85	86
*F*_1_-score	71	64	60	65
Balanced accuracy	87	74	72	76
TP^c^ improved atoms	100	100	97	92
FP^d^ falsified atoms	0	0	17	0
Hallucination rate	1	2	1	0
Hallucination detection	50	38	25	0
Inaccuracy rate	3	1	5	1
Inaccuracy detection	58	50	27	50

a Numbers represent the percentage values.

b PPV: positive predictive value.

c TP: true positive.

d FP: false positive.

**Figure 2.**
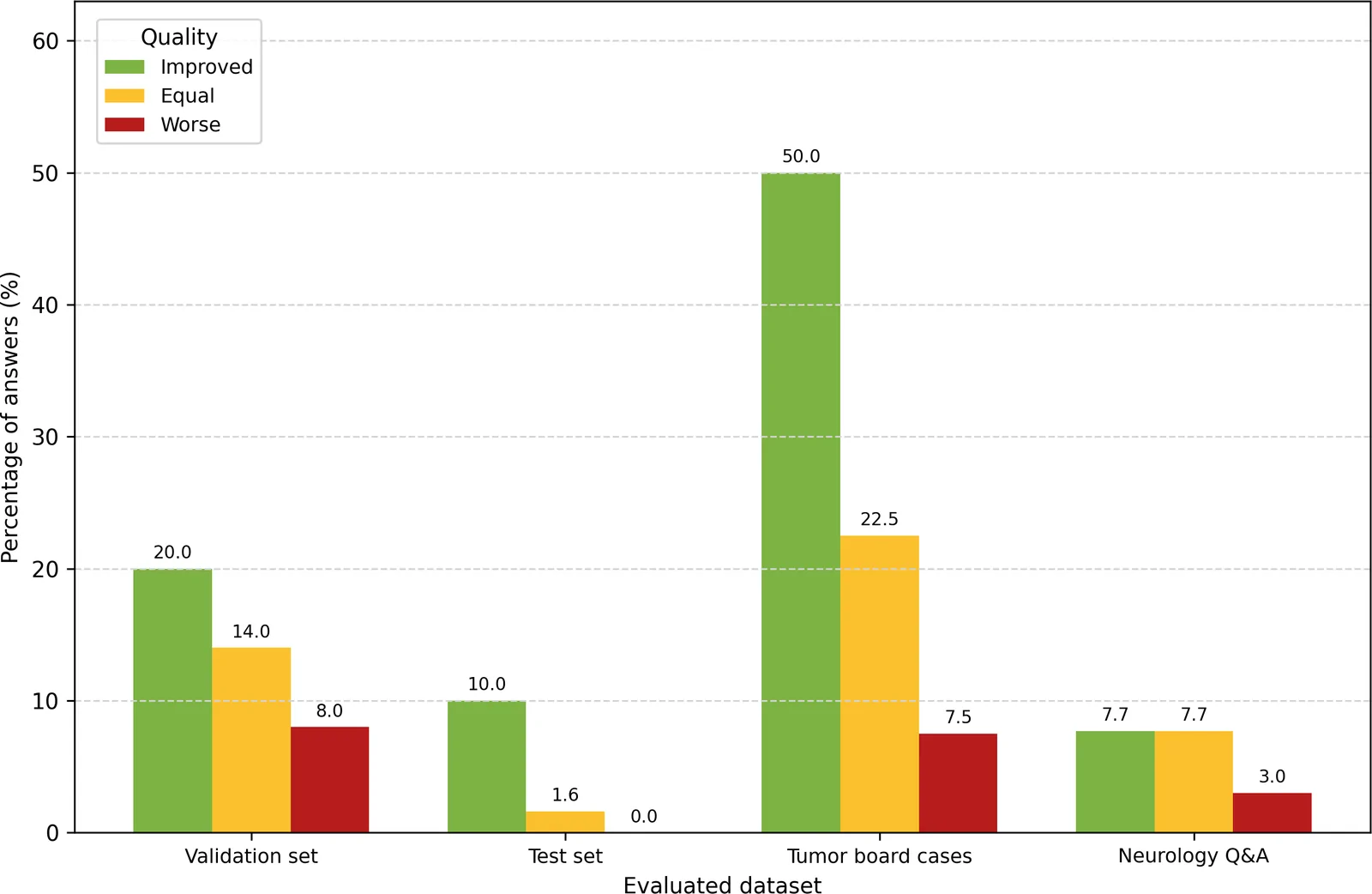
Change in final answer quality. We show how the overall answer quality changed by comparing the initial and final fact-checked answer. This is shown for our 4 evaluation question and answer (Q&A) datasets in 3 categories (improved, equal, and worse), as rated by human expert annotators. The figure displays changes by fact-checking; hence unchanged answers (no FALSE facts) are not included (validation set 56%, test set 88.3%, tumor board Q&A 20%, neurology Q&A 81.8%).

Overall answer improvements were seen in 20%, 10%, and 50% of cases in the validation, test, and tumor board–Q&A sets, respectively (Figure 2). Overall answer quality decreased in 8%, 0%, and 7.5 % of cases. The quality of the remaining answers remained the same.

Achieving results comparable to our own test set on the neurology Q&A shows that no overfitting occurred. Presenting with a sensitivity of 52%, a precision of 86%, and a balanced accuracy of 76%, the overall answer quality was improved, remained stable, or worsened in 7.7%, 7.7%, and 3% of cases, respectively (Figure 2).

**Figure 3.**
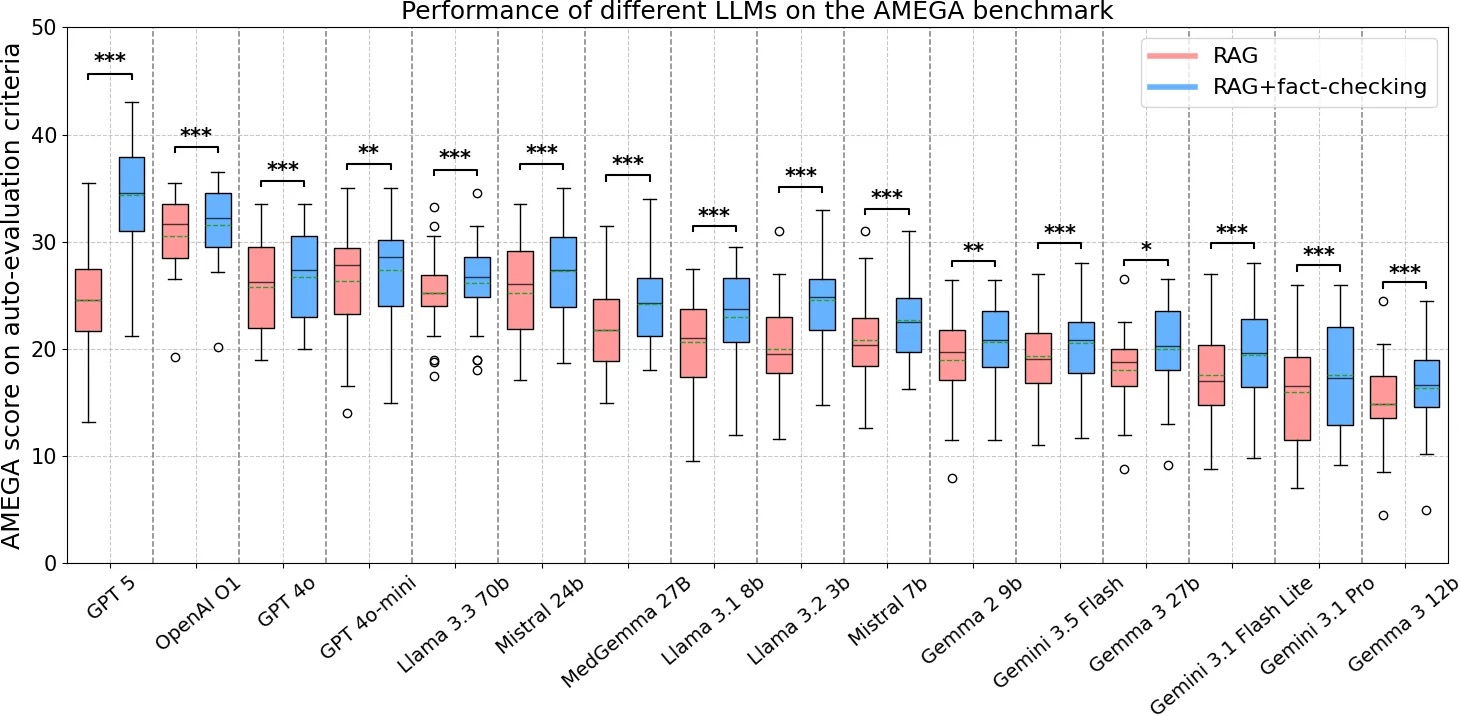
Results of auto-evaluation on the AMEGA (Autonomous Medical Evaluation for Guideline Adherence) benchmark. Scores for 16 different open-source and closed-source large language models (LLMs), with and without answer fact-checking. RAG: retrieval-augmented generation. ***P<.001, **P<.01, *P<.05.

**Figure 4.**
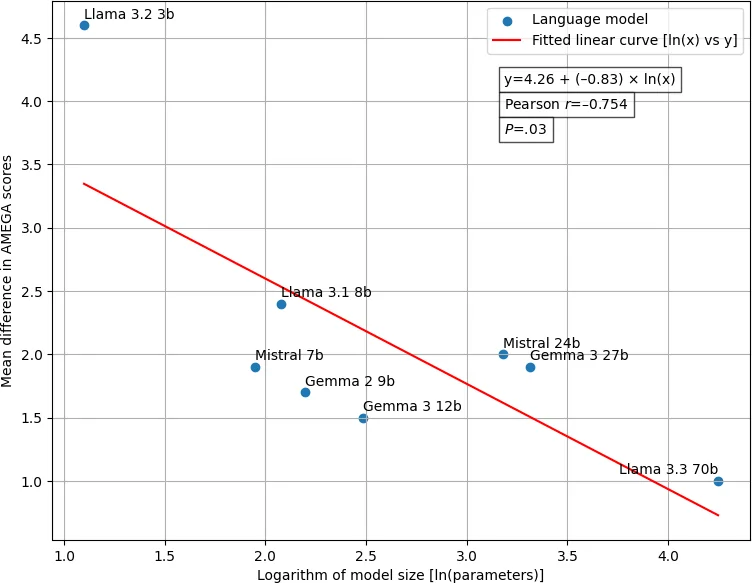
Results of auto-evaluation on the AMEGA (Autonomous Medical Evaluation for Guideline Adherence) benchmark. Fitted linear curve of the natural logarithm of large language model (LLM) parameter size, in billions, vs the average absolute score improvement from using fact-checking for that model.

The AMEGA auto-evaluation analysis revealed a significant improvement in answer quality by applying our fact-checking algorithm in contrast to RAG only for all LLMs tested (*P*<.001 for 13/16 models, *P*<.01 for 2/16 models, and *P*<.05 for 1/16 models; Figure 3; see Table S6 in Multimedia Appendix 1). The overall best answer quality (27.3) among nonreasoning models was achieved by GPT-4o-mini and Mistral 24B, after fact-checking was applied. The largest improvement with fact-checking was seen for Llama 3.2 3B. A significant negative correlation was found between the logarithm of the LLM model parameter size (in billions) and model improvement (Pearson correlation −0.754, *P*=.03; Figure 4). Even for the reasoning model OpenAI-o1, performance was significantly increased (*P*<.001), achieving the overall best model performance of 31.52.

### Explainability

An important aspect of the fact-checking framework is the explainability achieved by tracing each atomic fact to the most relevant chunk (passage) in medical guidelines. To assess this*,* we used the test-Q&A set and compared three similarity definitions of the best-fitting chunks selected from the vector database. A simple chain-of-thought prompt using GPT-4o identified the correct chunk in 75% of cases as the first choice and in 91.9% of cases among the top 3 chunks, outperforming a complex chain-of-thought prompt (instructing the LLM to assign scores and then rank) and cosine similarity with a text-embedding model (see Figure S4 in Multimedia Appendix 1 and Table S3 in Multimedia Appendix 1 for prompts).

### Open-Source Medical vs Nonmedical Models

In the comparison of open-source medical and nonmedical models (Table 2), MedGemma 27B performed best overall, with a balanced accuracy of 90%, a sensitivity of 83%, and a total improvement in answers of 30%, outperforming even the GPT-4o baseline. The high sensitivity score of MedGemma 27B was better than that of its Gemma 3 27B counterpart and GPT-4o (83% vs 46% vs 78%).

The 2 tested Qwen models showed comparable sensitivity (55% vs 56%), while Llama 3 70B considerably outperformed OpenBioLLM 70B (61% vs 33%). The same tendency holds true for their *F*_1_-scores and balanced accuracies. The positive predictive value was the highest for OpenBioLLM 70B; however, it had markedly reduced sensitivity and an elevated false-negative rate.

Looking at the improved overall answer quality as the main fact-checking outcome, MedGemma 27B demonstrated superior performance compared to its generalist counterpart, Gemma 3 27B, as well as the GPT-4o baseline (30% vs 20% vs 20%) and all other evaluated models. The Qwen models showed moderate results (generalist: 18% and medical: 24%), while Llama 3 surpassed OpenBioLLM (8% vs 4%). Among nonmedical models, Llama 3 achieved the highest balanced accuracy (79%).

**Table 2.** Comparing the fact-checking performance of multiple open-source large language models (LLMs) and the proprietary GPT-4o (the baseline used in previous experiments)^a^.

Evaluation metric^b^	GPT-4o (baseline)	Gemma 3 27B	MedGemma 27B	Llama 3 70B	OpenBioLLM 70B	Qwen 3 32B	Qwen 3 Medical 32B
TP^c^	6	6	11	12	7	8	8
FP^d^	3	3	3	2	1	2	3
TN^e^	89	84	84	78	79	83	82
FN^f^	2	7	2	8	14	6	7
Sensitivity (recall)	78	46	83	61	33	56	55
Specificity	96	97	97	97	99	98	96
Precision (PPV^g^)	66	68	79	83	88	80	72
*F*_1_-score	71	55	81	70	48	66	63
Accuracy	95	90	95	90	86	92	90
Balanced accuracy	87.3	71	90	79	66	77	76
TP improved atoms	100	100	91	98	100	82	89
FP falsified atoms	0	30	44	50	0	0	14
Overall answer quality improved	20	20	30	8	4	18	24
Overall answer quality equal	14	8	6	4	4	8	22
Overall answer quality worse	8	4	6	6	10	16	10
Hallucination rate	1	2	3	6	11	4	2
Hallucination detection	50	71	80	52	50	69	60
Inaccuracy rate	3	2	3	5	3	3	4
Inaccuracy detection	58	38	90	68	22	21	44

a The focus was on general-purpose vs medical fine-tuned models of different sizes: Gemma 3 27B vs MedGemma 27B; Llama 3 70B vs OpenBioLLM 70B; and Qwen 3 32B vs Qwen 3 Medical 32B.

b Numbers represent the percentage values.

c TP: true positive.

d FP: false positive.

e TN: true negative.

f FN: false negative.

g PPV: positive predictive value.

Results of LLM-as-a-judge automated rubric evaluation are shown for the baseline, GPT-4o, and the best-performing open-source model based on *F*_1_-score, MedGemma 27B, in Table 3. Results of all open-source models are available in Table S9 of Multimedia Appendix 1. Even though MedGemma 27B outperformed GPT-4o on the fact-checking task (atomic claim veracity prediction), GPT-4o scored better in four rubrics, MedGemma in one rubric, while scores were almost equal in two rubrics. While MedGemma improved more answers in total (30% vs 20%), certain aspects of its answers are still lacking compared to GPT. Most of MedGemma’s answers had significantly higher rubric scores after the fact-checking process was performed. When evaluating all LLMs together, context faithfulness was significantly improved (Table S9 in Multimedia Appendix 1).

**Table 3.** Comparison of large language model (LLM)–as-a-judge rubric scores^a^.

LLM or metric	Correctness, mean (95% CI)		Completeness, mean (95% CI)		Clarity, mean (95% CI)		Context faithfulness, mean (95% CI)		Coherence, mean (95% CI)		Medical harmfulness, mean (95% CI)		Calibration, mean (95% CI)	
Baseline or final^b^	Baseline	Final	Baseline	Final	Baseline	Final	Baseline	Final	Baseline	Final	Baseline	Final	Baseline	Final
GPT-4o	66 (60‐72)	66 (60‐73)	93 (91‐95)	92 (90-94)	87 (86‐88)	86 (85‐87)	88 (85‐91)	88 (84‐91)	89 (89‐90)	89 (89-90)	95 (92‐98)	94 (91‐97)	87 (86‐89)	87 (85‐89)
MedGemma 27B	57 (49‐65)	58 (50-65)	81 (76‐87)	86^c^ (82-91)	80 (78‐82)	83^c^ (81-85)	90 (87‐92)	92^c^ (91-93)	76 (71‐81)	80 (76-83)	94 (92‐95)	95^c^ (93-97)	86 (84‐87)	87^c^ (85-89)

a We show the final auto-evaluation scores for 7 rubrics, comparing the baseline model, GPT-4o, and the best-performing open-source model, MedGemma 27B. The scores are averaged across 50 answers on the validation set.

b Baseline scores are for initial answers based on retrieval-augmented generation (RAG). Final scores are for final answers after fact-checking was performed.

c *P*<.05.

### Baseline Comparison

Comparison with the competing self-correcting framework self-refine [17] on AMEGA is shown in Table 4. The improvement rate using self-refine depended on the model evaluated. The highest rate was seen for GPT-4o, while Gemini models and open-source models showed lower performance improvements. In comparison with self-refine, fact-checking was significantly better for two smaller open-source models, while there was no difference for recent Gemini models. For GPT-4o, self-refine showed significantly better performance.

**Table 4.** Comparison of our fact-checking and the self-correcting self-refine approaches using AMEGA (Autonomous Medical Evaluation for Guideline Adherence)^a^.

Model	Initial score on AMEGA	With fact-checking (our approach)	With self-refine [17]	Difference fact-checking to self-refine
GPT-4o	25.4	26.5 (+1.1)^b^	28.3 (+2.9)^b^	–1.8^c^
GPT-4o-mini	26.3	27.3 (+1.0)^b^	28.0 (+1.7)^b^	–0.7
Gemini 3.1 Pro	16.0	17.6 (+1.6)^b^	17.5 (+1.5)^b^	+0.1
Gemini 3.1 Flash Lite	17.6	19.7 (+2.1)^b^	19.1 (+1.5)^b^	+0.6
Gemini 3.5 Flash	19.3	20.5 (+1.2)^b^	20.0 (+0.7)^b^	+0.5
Gemma 3 27B	18.1	20.0 (+1.9)^b^	19.4 (+1.3)^b^	+0.6
MedGemma 27B	21.8	24.2 (+2.4)^b^	22.8 (+1.0)^b^	+1.4^c^
Llama 3.2 3B	20.2	24.6 (+4.4)^b^	21.0 (+0.8)^b^	+3.6^b^

a We show the final auto-evaluation scores on AMEGA for five representative large language models (LLMs) based on (1) initial responses; (2) responses corrected with our fact-checking framework; and (3) responses corrected with the competing self-refine approach.

b *P*<.001, compared with the initial score.

c *P*<.05.

## Discussion

Our study introduces the first application of an atomic fact-checking framework designed to enhance the reliability and explainability of LLMs used in medical Q&A. By decomposing LLM-generated responses into discrete atomic facts and rigorously verifying each against an authoritative vector database, the framework significantly reduced hallucinations and inaccuracies. Medical expert assessment and automated benchmarks demonstrated notable improvements in factual accuracy, achieving up to a 50% overall answer improvement and an 80% hallucination detection rate. Additionally, the framework achieved high explainability by tracing each atomic fact back to the most relevant chunks from the database, providing a granular, transparent explanation of the generated responses. Rubric-based autoevaluation not only revealed an LLM dependency in score improvement but also significantly increased source faithfulness across all LLMs. Compared with the self-refine approach, performance gains differed depending on the model chosen. While the former GPT-4o frontier model profited from self-refinement, there was no difference for current Gemini models, and smaller open-source models significantly benefited from atomic fact-checking.

Our framework increased the factual accuracy and overall quality of LLM-generated responses. Numerical hallucinations, such as incorrect drug dosages, were frequently identified and corrected, as were entity hallucinations, such as the conflation of different treatment procedures. In a small proportion of cases (8%, 0%, 7.5%, and 3% across Q&A sets), the answer quality declined due to the retrieval of less relevant chunks or to potential hallucinations introduced during the correction process. However, since the proportion of answers that improved (20%, 10%, 50%, and 7.7%) was substantially higher, the overall effect of fact-checking clearly enhanced the answer quality. Different degrees of improvement come down to the varying complexity of questions across the datasets and to different rates of atomic facts being identified as FALSE and corrected.

Notably, the observed gain was strongest in real tumor-board questions. The most challenging and clinically realistic dataset achieved the highest rate of answer improvement (50%). The other 3 Q&A datasets (validation, test, and neurology-Q&A sets), where questions were straightforward and usually answerable directly from guideline passages, resulted in moderate improvement after fact-checking (20%, 10%, and 7.7%). Owing to this close correspondence, the potential for further improvement through fact-checking is inherently limited. In contrast, the tumor board cases were substantially more complex and based on real-world patient scenarios rather than guideline excerpts. Consequently, there was no exact blueprint answer available in the source material, leaving greater room for iterative refinement and revision of responses. This suggests that the system contributes most when queries are complex and multifactual, for example, in clinical settings.

Regarding the performance of medical fine-tuned LLMs in detecting incorrect facts, the model size appeared to be the most influential factor. As smaller models are less capable of answering complex questions, they derive great benefit from post hoc fact-checking (Figure 4). Fine-tuning for the medical domain can boost task specialization, but the effect is strongly dependent on individual details, differing from model to model and from adaptation to adaptation, and making direct comparison difficult. We observed that medical fine-tuned models mostly had worse performance, with MedGemma 27B marking an exception. Hence, it cannot be said that medical fine-tuned models show superior performance and improvement in general.

MedGemma 27B achieved notable results and improvements through fact-checking, outperforming even the GPT-4o baseline. Being a reasoning model (it outputs “thinking” tokens before the final response) likely helps its performance. Reasoning models also have the additional benefit of providing more interpretable answers. Additionally, MedGemma may have benefited from more effective fine-tuning compared with the other models.

Consistently, the answer quality of MedGemma 27B’s answers improved according to LLM-as-a-judge evaluation metrics after fact-checking was applied. Nevertheless, its performance remained below that of the proprietary GPT-4o model, indicating room for improvement.

RAG-based medical chatbots may qualify as Class IIa medical devices according to the European Medical Device Regulation (MDR) [3]. Trustworthiness, the combination of explainability, traceability, and transparency, is a key prerequisite under the MDR [20]. Although LLMs inherently explain their responses, these justifications can be misleading. Pure RAG remains susceptible to intrinsic limitations of LLMs, such as the incorporation of unsupported internal knowledge or incorrect synthesis of information across retrieved chunks. Thus, retrieval augmentation alone cannot fully guarantee factual consistency [21,22]. Atomic fact-checking serves as an explicit verification layer, systematically validating the generated claims, thereby improving factual reliability beyond what RAG alone can achieve [23]. LLMs improving over time might alleviate these issues, but, as they will always be stochastic, a rigorous verification mechanism remains necessary, especially in safety-critical domains such as medicine.

The fact-checking framework provides an additional advantage beyond answer refinement alone: it enables fact-wise verification and traceability of the generated statements back to the supporting source documents. This fine level of explainability and evidence grounding is another strength of the framework. Importantly, this process is outsourced from the internal reasoning of LLMs and directed toward a potential user. While our current work does not constitute a complete analysis of trustworthiness in all its dimensions, findings from a randomized controlled setting [24] provide relevant evidence: presenting AI-generated recommendations decomposed into individually verifiable claims and linked to source guidelines, thereby presenting a practical application of the fact-checking algorithm, was associated with substantially higher clinician trust than traditional explainability approaches.

While the self-refine method achieved similar improvements in answer quality in larger models (greater for GPT models but lower for Gemini models), we demonstrated that the benefit of our framework was greater in smaller LLMs. Smaller models achieved higher improvements using the fact-checking framework on benchmarks, including AMEGA, where our approach also outperformed the competing single-pass correction baseline. The even higher usability of the fact-checking algorithm in smaller LLMs is especially interesting, considering scenarios of potential on-premises deployment in medical institutions. The fact-checking approach offers explainability advantages that self-refine lacks. The generally lower Gemini scores may stem from prompts being optimized for the GPT model family and, for consistency, were used for all experiments.

Our work has several limitations. As we sought to evaluate open Q&A capabilities, we designed novel evaluation datasets. Our evaluation included 215 human-evaluated Q&As that were supplemented by autoevaluations using the AMEGA benchmark with 1337 scoring elements. Altogether, this corresponds to 1552 evaluated question-answer instances used in this study. This number is limited, and larger scale validation could further strengthen generalizability; however, the size of our manually evaluated datasets is comparable to that of many prior studies in the medical AI domain, where human expert annotation and review require substantial time and resources. Nevertheless, evaluation on a large-scale public benchmark is an important future step. Furthermore, all pipeline steps rely on LLM generation. It is possible that errors can propagate from one step to another. Future work could explore approaches to make the process more rigorous, such as Graph-RAG techniques for grounding data into graph structures for more robust generation. Finally, the entire fact-checking and rewriting process uses around 10 times more tokens than just the initial response generation (as shown in Table S11 of the Multimedia Appendix 1), which increases the cost and the latency of the system. A more token-efficient approach for our application is currently under investigation.

But still, in a world of rapidly generated and propagated data, it is more important than ever to check your facts thoroughly.

To conclude, we present the application of an atomic fact-checking algorithm that identifies factual inaccuracies and hallucinations in medical Q&A. Correcting these findings improves the overall answer quality while achieving fact-wise explainability, paving the way for more credible clinical use of LLMs.

## Supplementary material

10.2196/92090 Multimedia Appendix 1 Prompts, few-shot examples, and further experiments for atomic fact-checking in medical retrieval-augmented generation systems.

10.2196/92090 Checklist 1 TRIPOD-LLM checklist.

## Abbreviations

AMEGA: Autonomous Medical Evaluation for Guideline Adherence
LLM: large language model
MDR: Medical Device Regulation
Q&A: question and answer
RAG: retrieval-augmented generation
TRIPOD: Transparent Reporting of a Multivariable Prediction Model for Individual Prognosis or Diagnosis
